# Development and validation of the screening tool for identifying elder abuse by caregivers (STIEAC)

**DOI:** 10.1371/journal.pone.0351005

**Published:** 2026-06-17

**Authors:** Oludoyinmola Omobolade Ojifinni, Obioma C. Uchendu

**Affiliations:** 1 School of Clinical Medicine, Faculty of Health Sciences, University of the Witwatersrand, Johannesburg, South Africa; 2 Department of Community Medicine, College of Medicine, University of Ibadan, Nigeria; Jawaharlal Institute of Postgraduate Medical Education and Research, INDIA

## Abstract

**Purpose:**

Elder abuse is a global concern, yet detection methods focus mainly on victims. In community settings, trusted caregivers may perpetrate abuse that remains hidden due to cultural norms. Existing tools lack a contextual fit for sub-Saharan Africa. This study developed and validated the Screening Tool for Identifying Elder Abuse by Caregivers (STIEAC) in Southwest Nigeria.

**Methods:**

The study used Artino’s seven-step questionnaire development framework, combining a literature review and caregiver focus groups to create culturally relevant items across the neglect, physical, psychological, financial, and sexual abuse domains. Expert panels ensured content and face validity, followed by pilot testing after which the revised tool was administered in a survey among 1119 caregivers. The psychometric evaluation included factor analysis, reliability (Cronbach’s alpha, Guttman split-half), and construct validity via correlations with the Zarit Burden, General Health Questionnaire-12, and Katz Activities of Daily Living scales.

**Results:**

The STIEAC’s 10-item structure encompasses the five elder abuse domains, demonstrating high internal consistency (Cronbach’s α = 0.90) and reliability (split-half coefficient = 0.713). Bartlett’s test was significant (χ² = 7642.08, p < 0.001), and the KMO measure was adequate (0.896). Domain-specific reliability was highest for neglect (α = 0.87) and lowest for sexual abuse (α = 0.03). Construct validity was supported by a positive correlation with the ZBI (r = 0.294, p = 0.001) and negative correlations with the GHQ-12 (r = −0.349, p = 0.001) and the Katz ADL (r = −0.160, p = 0.001).

**Conclusion:**

The STIEAC is a reliable 10-item tool reflecting culturally nuanced elder abuse perpetration by caregivers in community settings. Despite the low consistency of the sexual abuse subscale, it has strong overall validity, filling a critical gap for screening for elder abuse in sub-Saharan Africa and assisting researchers and practitioners.

## Introduction

Screening for elder abuse often focuses on older people, and several tools for the identification of older persons suffering abuse have been developed and validated in different contexts [1–3]. While there are tools that caregivers can use in home settings, others depend on self-reports of abuse with a focus on the presence of the risk of abuse indicators or the identification of signs of abuse by a professional [1,3]. The identification of older persons who have suffered or are suffering from abuse is, however, often difficult in institutions and healthcare settings, particularly because perpetrators are in a position of trust relative to the victims [4,5]. Although the proportion of cases identified in these settings is seemingly high, it is still the tip of the iceberg when compared with prevalence studies on elder abuse. Whereas some studies show that older persons report experiences of abuse, other studies reveal that older persons are reluctant to report abuse, while in some instances, third-party observers are more likely than older people are to report elder abuse [6–8]. There are many obstacles that prevent older people who experience abuse from being identified. These include the perception among older persons and their caregivers that abusive situations are private family matters and must be dealt with as such; fear of retaliation, shame or stigma; professionals within healthcare settings being unaware or untrained on how to screen for and identify elder abuse; and the belief that there are specific personnel who should handle abusive situations, among others [9–15]. Discrepancies in the reporting of elder abuse have also been documented when reports among older persons are compared with caregiver reports [16,17].

The accuracy of the reporting of elder abuse can be improved through the involvement of multiple informants, including older persons, their caregivers and health care providers [16]. The possibility exists that caregivers may not consider their actions abusive until they see such actions perpetrated by other people [18]. Some caregivers may also unwittingly perpetrate abuse as an unconscious response to the burden of care they experience or in response to an unwanted action in the older person [19]. Screening the caregivers of older persons for the possibility of abuse perpetration is a way of providing one lens to guide the identification and provision of context-specific interventions for elder abuse.

Research has been conducted on the burden of care experienced by caregivers of older persons who have some medical illness or cognitive dysfunction [19–21]. Studies have also compared older persons’ reports of abuse with their caregivers’ self-reports of abuse in healthcare and institution settings [16,17,22]. However, there is a paucity of information on the perpetration of elder abuse from the perspective of caregivers who provide care to older persons who have no physical or cognitive impairment beyond the influence of aging within community settings. Tools that have been used for research within healthcare settings and institutions may not be applicable at the community level or easily applied without some specialized training. Existing tools include the Hwalek-Sengstock Elder Abuse Screening Test (H-S/EAST), and the self-report Vulnerability to Abuse Screening Scale (VASS) which are self-report tools administered to older adults to capture vulnerability, dependence and coercion [23]. Although both tools are easy to administer and can be used in community settings, both tools show inconsistent internal reliability where H‑S/EAST has an α ∼0.29 and VASS subscales α range from ∼0.31 to 0.74 [1,24]. Cohen’s 10-item Self-Disclosure Tool, the Caregiver Abuse Screen (CASE) and the Brief Abuse Screen for the Elderly (BASE) are administered to informal caregivers in home or community settings by health and social care professionals. While CASE has good construct validity and acceptable internal consistency (α ∼0.71) and BASE shows high predictive validity (>85%), both require training and are best used in tandem with more detailed assessments [1,3,24]. The Elder Abuse Suspicion Index (EASI), is a brief, practitioner‑administered tool designed for cognitively intact older adults; it is applied by physicians or nurses directly to older patients during consultations and combines five direct questions to the older person with one clinician‑judgment item. EASI has moderate sensitivity (≈0.44–0.47) and good specificity (≈0.75–0.77), making it useful for ruling out abuse and prompting referral rather than definitive diagnosis [1,3,24].

Furthermore, there are no documented tools for assessing caregiver perpetration of elder abuse in southwest Nigeria. Therefore, in this study, we developed and validated the Screening Tool for Identifying Elder Abuse by Caregivers (STIEAC). This tool will be appropriate for detecting perpetration of elder abuse among caregivers of older persons at the community level.

## Methods

### Study design

An exploratory sequential mixed-method design was employed in this study. We used a modification of the seven steps for designing and validating a questionnaire described by Artino and colleagues [25]. Step 1 involves determining the constructs to be examined by the questionnaire and identifying existing tools covering the specified constructs. Step 2 involves focus group discussions or interviews to explore the perceptions of the constructs among the population of interest. In step 3, the findings from the literature are synthesized with the FGD findings to reconcile the definitions. This is followed by the development of survey items to populate the questionnaire in step 4. Expert review of the questionnaire to assess content validity is performed in step 5, followed by cognitive interviews to assess potential respondents’ understanding of the survey items (step 6). Pilot testing on a sample of the expected respondents is step 7, and the results are analyzed to determine the validity and reliability of the tool [25].

We conducted a literature review in which we identified existing tools that have been used previously among caregivers of older persons in settings different from ours. The constructs identified from these tools included neglect and physical, psychological/emotional, financial and sexual abuse. Following a modification of the survey items for cultural acceptability, we had expert validation of the items in a workshop setting where a group of public health researchers with expertise in questionnaire design reviewed the questionnaire for face and content validity. Modifications for the removal of ambiguity in the questionnaire items were suggested and implemented. Two other researchers with expertise in elder abuse research subsequently reviewed the questionnaires, and further modifications were made to the items where needed. This was followed by a qualitative exploration of the perceptions of elder abuse, its perpetration, perpetrators and prevention involving focus group discussions (FGDs) conducted among purposively selected adult caregivers of older persons. The views expressed were used to further modify the questionnaire items for cultural relevance to the context prior to the quantitative study. The pre-final questionnaire was pilot-tested in a cross-sectional survey among 106 adult caregivers of older persons selected via multistage sampling. Further details of the validation process are presented in subsequent sections.

### Study setting

The study was performed in two local government areas (LGAs), one rural area (Afijio), and one urban area (Ibadan South-East), selected by systematic random sampling in Oyo State, Nigeria. Oyo State is one of the southwestern states and consists of 33 LGAs, of which nine are peri-urban, 12 are urban and 12 are rural. The rural LGAs consist of agricultural communities where the occupation is predominantly farming. The peri-urban LGAs are a mixture of urban areas and agricultural communities.

### Study population and sampling

The study population included adults aged 18–59 years who provided care for older persons. Participants were recruited in March 2016. To be included in the study, the caregivers had to be employed to care for an older person as the primary caregiver or live with and care for an older person in the same house. A purposive sample of caregivers meeting the inclusion criteria was recruited for the qualitative study while multistage sampling was used for the quantitative aspect of the study. Using the sampling frame of rural and urban LGAs in Oyo State, two LGAs, one rural area (Afijio in Oyo senatorial district), and one urban area (Ibadan South-East, one of the core LGAs within the Ibadan metropolis), were selected from the 12 urban and 12 rural LGAs by balloting. The 12 wards in Ibadan South-East LGA are divided into predominantly residential and predominantly commercial with few residential houses. Two wards each were selected from each of the groups giving a total of four wards in the urban LGA. One ward was selected from the 10 wards in Afijio LGA using simple random sampling. Participant selection was extended to the contiguous ward to complete the sample size. Using cluster sampling, all households in the selected wards were approached to identify eligible adults. All adults who consented to participate in the study were interviewed.

### Tool development and validation

The development of the STIEAC began by modifying the “*Prevalence of Elder Abuse Survey questionnaire”* [26] and the *“Caregiver questionnaire for the report of Elder Abuse in the Family in Spain”* [27]. The *Prevalence of Elder Abuse Survey questionnaire* [26] is an interviewer-administered tool that assesses older persons’ experience of abuse perpetrated by any adult, including relatives, neighbors or paid caregivers. A study comparing self-reported perpetration of abuse by caregivers with older persons’ experience of abuse conducted in Spain used the *caregiver questionnaire for the report of elder abuse in the family in Spain* [27]. The tool was translated into Yoruba, the local language, and back-translated to ensure consistency. Face and content validation of the merged tool was performed during a workshop where a panel of public health researchers read and discussed the questions. During the workshop, the study methodology was refined and some of the questions were rephrased for applicability in community settings. Following the panel, two experts who had conducted studies on abuse among older persons in Oyo State reviewed the contents of the questionnaire, assessing coverage of essential elements of elder abuse. The resulting questionnaire was further modified following analysis of the qualitative aspect of the study. Modifications, including the addition of new themes and rephrasing of the questions to ensure improved cultural acceptability of the survey tool was done using information obtained from the qualitative data analysis. The themes in the initial instrument were neglect (four questions), physical (six questions), psychological (three questions), verbal (one question), financial (four questions) and sexual abuse (two questions), resulting in a total of 20 questions. The tool was then pilot tested among 106 caregivers in two LGAs, one rural (Ibarapa Central) and one urban (Akinyele), which were selected due to their similarity to the LGAs selected for the study. Principal component analysis (PCA) was done on the different types of elder abuse. Factors whose loading on PCA were less than 0.4 were deleted from the questionnaire. This resulted in a final set of 10 questions which were then administered in the full survey. Reliability analysis on these 10 questions gave an overall Cronbach’s alpha of 0.90 with the section on neglect having the highest reliability (Cronbach’s α 0.87) whereas the sexual abuse section, with a Cronbach’s α of 0.03 had the lowest reliability (Table 1). The section on sexual abuse was however retained in the full survey so as not to lose the construct entirely.

**Table 1:** Reliability analysis following the pilot test.

S/N	Questionnaire segments	Cronbach’s alpha
1.	Perpetration of elder abuse *(Overall)*	0.90
1.	Neglect	0.87
2.	Physical abuse	0.77
3.	Emotional/Psychological abuse	0.85
4.	Financial abuse	0.71
5.	Sexual abuse	0.03

### Data management and analysis

#### Qualitative data.

The interview guide for the qualitative aspect of the study included questions on what participants believed elder abuse to be, how they would identify older persons who suffered abuse, opinions about possible perpetrators of abuse and views on prevention of elder abuse. The guides were developed in English, translated into Yoruba, the local language, and back-translated into English to ensure consistency of meaning. A pre-test of the guide was performed, and ambiguous questions were deleted or rephrased. All the FGDs and IDIs were facilitated by the first author in Yoruba and were digitally recorded, translated and transcribed. The transcripts were imported into Atlas.ti version 6.0.16 for thematic analysis, which included a hybrid of inductive and deductive coding [28,29]. The themes derived from the data on the definitions and types of elder abuse guided the modification of the questionnaire for the quantitative aspect of the study. Details of the qualitative study have been presented in full elsewhere [30].

#### Quantitative data.

The data obtained were analyzed using SPSS version 22. Exploratory factor analysis of the burden data was conducted using principal component analysis and varimax rotation to determine the factorial structure of the data. The adequacy of the sample was evaluated using the Bartlett test of sphericity and the Kaiser-Meyer-Olkin (KMO) test. Factors whose loading on PCA was less than 0.4 were excluded from the instrument. Internal consistency was determined with Cronbach’s alpha, where a Cronbach’s α of at least 0.70 was accepted as indicating good internal consistency [31,32]. The instrument reliability was also tested with the Guttman split-half correlation, a correlation coefficient calculated between the scores on two halves of the test. A high value >0.70 implies good reliability [32]. The consistency of the items in the scale was assessed using item total correlations. Construct validity was assessed using Spearman’s correlation coefficients between the STIEAC and the Katz functional activities of daily living (Katz ADL) [33], the modified, short version of the Zarit burden interview (ZBI) [31] and the GHQ-12 [34]. The modified, short version of the Zarit burden was validated prior to its use in this study [35].

### Ethical considerations

The study was conducted in accordance with the ethical standards as laid down in the 1964 Declaration of Helsinki and its later amendments. Participation in the study was voluntary. All the participants were provided with an information sheet that provided details about the study and the implications of participation, and any questions raised were answered to the satisfaction of the individuals. Consent forms were signed by everyone who agreed to be included in the study. No identifying information was collected, and all the data generated were saved in a password-enabled computer accessible only to the authors. Ethical approval was obtained from the Oyo State Ministry of Health Ethics Committee (Clearance number: AD 13/479/794).

## Results

### Sociodemographic characteristics

A total of the 1119 participants were interviewed in the quantitative survey. The details of the participants’ sociodemographic characteristics are shown in Table 2.

**Table 2:** Sociodemographic characteristics of the study participants.

Variable	Frequency (%) N = 1119
**Age (years)**	*Mean age = 38.63 ± 8.72*
18–24	49 (4.4)
25–44	508 (45.4)
≥ 45	562 (50.2)
**Sex**	
Male	450 (40.2)
Female	669 (59.8)
**Marital status**	
Single	242 (21.6)
Married	877 (78.4)
**Occupation**	
Civil servant	56 (5.0)
Student	57 (5.1)
Teaching	99 (8.8)
Farming	107 (9.6)
Artisan	280 (25.0)
Trading	520 (46.5)
**Level of education**	
No formal education	56 (5.0)
Completed primary	212 (18.9)
Completed secondary	600 (53.6)
Completed tertiary	251 (22.4)
**Religion**	
Christianity	646 (57.7)
Islam	441 (39.4)
Traditional	30 (2.7)
Other	2 (0.2)
**Tribe**	
Yoruba	1071 (95.7)
Igbo	32 (2.9)
Hausa	13 (1.2)
Other	3 (0.3)
**Region**	
Rural	556 (49.7)
Urban	563 (50.3)

### Qualitative

The participants in the FGDs, which constituted the qualitative aspect of the study, provided descriptions consistent with the types of abuse documented in the literature. The descriptions provided were integrated into the STIEAC to ensure the cultural applicability of the tool. While the participants described actions consistent with verbal abuse, there was no reference to sexual abuse in the discussions (Table 3).

**Table 3:** Sample quotes from the qualitative data.

Types of elder abuse	Sample quotes
Physical abuse	*“…she is on the wheel chair, she will be locked in in the morning when everyone is going out, and the door will not be opened again for her till later in the evening when we are back, since she cannot do anything for herself, she will be left to sit in the wheel chair till everyone comes back…”* – Female urban FGD participant (40–59 years)
Verbal abuse	*“…some people will be shouting at their older person … there is no need to shout at them, we should know how to talk to them so that they will be happy too…”* – Female urban FGD participant (40–59 years)
Neglect	*“...some people who have older persons at home do not take care of the older person...”* – Male rural FGD participant (40–59years)
Financial abuse	*“For instance, because an old woman cannot see well, when the woman calls a child to buy kerosene, the child can take the woman’s money and give her the wrong amount as change, keeping the money for himself. That kind of behavior is not good …”* – Female urban FGD participant (31–49years)

### Quantitative

The final instrument (Table 4; Supplement 1) included 10 questions covering neglect (three questions), physical (two questions), psychological (two questions), financial (two questions) and sexual abuse (one question). These questions were analyzed for reliability and validity.

**Table 4:** The Screening Tool for Identifying Elder Abuse by Caregivers (STIEAC).

Item	Abuse type	Statement	Code
1.	Neglect	Would you say you have ever been indifferent to his/her nutritional desires?	
2.		Do you think you have ever been unconcerned about his/her clothing?	
3.		Would you say you have ever been unconcerned regarding his/her hygiene?	
4.	Physical	Have you ever restricted him/her with some mechanism that prevented them from moving about freely, locked them up or put them in a chair from which they could not get up without help?	
5.		Have you ever given him/her non-prescribed pharmaceutical drugs for any reason at all?	
6.	Psychological/Emotional	Have you ever failed to care for their affective or emotional needs?	
7.		Have you ever intimidated or threatened them in any way?	
8.	Financial	Have you ever forced them to sign documents or change their will?	
9.		Have you ever faked their signature?	
10.	Sexual	Have you ever engaged in sexual activity with them without their consent?	

**Instructions**: Answer the following questions concerning your care of the older person for whom you are responsible using the codes: ***1*** *= Once in the past year;* ***2*** *= Twice in the past year;* ***3*** *= Approximately 5 times in the past year;* ***4*** *= 6–10 times in the past year;* ***5*** *= More than 10 times in the past year;* ***6*** *= Not in the past year, but it has happened before;* ***7*** *= This has never happened.*

### Reliability

The result of the reliability analysis for the STIEAC was a Cronbach’s α of 0.912 and a Guttman split-half correlation coefficient of 0.713 being minimum score for acceptable reliability. The results of the reliability analysis for the sections of the tool revealed an improvement in the reliability of the financial abuse section with a Cronbach’s α of 0.91 while the physical abuse section improved from 0.77 to 0.81 (Table 5). The Cronbach’s α of the sexual abuse section was not determined as only one item remained following deletion of items with factor loadings <0.4.

**Table 5:** Reliability coefficients for the STIEAC.

S/N	Questionnaire segments	Cronbach’s alpha	
		Pilot	Full survey
2.	Perpetration of elder abuse *(Overall)*	0.90	0.91
6.	Neglect	0.87	0.87
7.	Physical abuse	0.77	0.81
8.	Emotional/Psychological abuse	0.85	0.76
9.	Financial abuse	0.71	0.91
10.	Sexual abuse	0.03	

The mean scores of the individual items in the scale ranged from 5.77 to 6.82, and the corrected item–total correlation was positive (>0.50) for all the items in the scale. Withdrawal of any of the items did not improve the internal consistency of the scale (Table 6).

### Construct validity

Assessment of the correlation of the STIEAC with other instruments (Table 7) with Spearman’s correlation (rs) revealed a significant positive correlation with the Zarit Burden Interview (ZBI). However, there was a negative correlation with the GHQ-12 and the Katz assessment of Activities of Daily Living (ADL) for the care recipients.

**Table 6:** Mean score for items and item-total (corrected) correlation in the STIEAC.

Item description	Mean ± SD	Corrected item total correlation	Cronbach’s α if deleted
**1.** Would you say you have ever been indifferent to his/her nutritional desires?	5.77 ± 2.30	0.653	0.895
**2.** Do you think you have ever been unconcerned about his/her clothing?	6.06 ± 2.01	0.731	0.885
**3.** Would you say you have ever been unconcerned regarding his/her hygiene?	6.19 ± 1.89	0.733	0.884
**4.** Have you ever restricted him/her with some mechanism that prevented them from moving about freely, locked them up or put them in a chair from which they could not get up without help?	6.37 ± 1.64	0.678	0.888
**5.** Have you ever given him/her non-prescribed pharmaceutical drugs for any other reason at all?	6.25 ± 1.74	0.721	0.885
**6.** Have you ever failed to care for their affective or emotional needs?	6.49 ± 1.54	0.708	0.886
**7.** Have you ever intimidated or threatened them in any way?	6.60 ± 1.36	0.618	0.892
**8.** Have you ever forced them to sign documents or change their will?	6.76 ± 1.10	0.634	0.893
**9.** Have you ever faked their signature?	6.78 ± 1.07	0.617	0.894
**10.** Have you ever engaged in sexual activity with them without their consent?	6.82 ± 0.93	0.622	0.895

### Factor analysis

The Bartlett test of sphericity was statistically significant (χ^2^ = 7642.08, *df* = 45, p < 0.001) while the Kaiser-Meyer-Olkin measure of sample adequacy was 0.896, an excellent justification for the factor analysis. Using principal component analysis with orthogonal rotation using a varimax approach for easy interpretation of the factor loadings, two components (factors) whose eigenvalues were greater than 1.0 were extracted (Table 8). These components accounted for 71.45% of the total item variance, with the first component accounting for 55.80% of the total item variance and the second component accounting for 15.65% (Table 8).

**Table 7:** Correlations of the STIEAC with other instruments.

Other instruments	Correlation with abuse perpetration tool	p value
The 12-item Zarit Burden Interview (ZBI)	0.294	<0.001
The General Health Questionnaire (GHQ-12)	−0.349	<0.001
Katz assessment of Activities of Daily Living (ADL)	−0.160	<0.001

**Table 8:** Principal component analysis results.

No	Item description	Factor loadings	
		1	2
1.	Would you say you have ever been indifferent to his/her nutritional desires?	0.688	0.42
2.	Do you think you have ever been unconcerned about his/her clothing?	0.745	0.478
3.	Would you say you have ever been unconcerned regarding his/her hygiene?	0.768	0.32
4.	Have you ever restricted him/her with some mechanism that prevented them from moving about freely, locked them up or put them in a chair from which they could not get up without help	0.724	0.314
5.	Have you ever given him/her non-prescribed pharmaceutical drugs for any other reason at all?	0.766	0.295
6.	Have you ever failed to care for their affective or emotional needs?	0.778	0.035
***7.***	Have you ever intimidated or threatened them in any way?	0.727	−0.286
8.	Have you ever forced them to sign documents or change their will?	0.767	−0.517
9.	Have you ever faked their signature?	0.75	−0.51
10.	Have you ever engaged in sexual activity with them without their consent?	0.752	−0.512

## Discussion

This study presents the development and validation of the Screening Tool for Identifying Elder Abuse by Caregivers (STIEAC), which is designed for use in community settings to detect elder abuse perpetrated by caregivers. Whereas existing tools have been used in healthcare and other institutional settings [19–21] to identify victims of elder abuse [1], the STIEAC fills the significant gap in tools applicable outside these settings, especially within the cultural context of Southwest Nigeria. The STIEAC demonstrated strong internal consistency, with a Cronbach’s alpha of 0.90 and a Guttman split-half coefficient of 0.71, indicating reliable performance of the instrument in measuring elder abuse perpetration across various abuse domains. This reliability is supported by positive item-total correlations, suggesting that each item contributes meaningfully to the overall construct of caregiver-perpetrated elder abuse. Furthermore, the qualitative component of the tool development contextualized the instrument items and revealed important perceptual insight such as recognition of neglect, verbal abuse, and financial exploitation, which substantiate the cultural relevance of the tool. The framing of the questions in a non-accusatory and culturally sensitive manner when used in a self-assessment context with assurance of confidentiality will enhance early detection of abusive behavior.

Construct validity was supported by significant correlations with established measures such as the Zarit Burden Interview (ZBI), the General Health Questionnaire (GHQ-12) and the Katz Activities of Daily Living (ADL). The positive correlation with the ZBI and the negative correlations with the GHQ-12 and Katz ADL align with theoretical expectations that caregiver burden correlates positively with abusive behaviors, whereas caregiver mental health and the care recipient’s functional independence are inversely related. This underscores the appropriateness of the STIEAC for screening for abuse risk in caregivers experiencing burden and stress while also caring for relatively functional older persons. The burden of care experienced by caregivers and mental health problems such as stress and anxiety can place caregivers at increased risk of perpetrating elder abuse [36–39]. Psychological distress, personality and mental health problems like anxiety or depression among caregivers, as measured by the GHQ‑12, is consistently associated with increased perpetration of elder abuse, with community‑based evidence demonstrating a significant positive correlation between GHQ‑12 scores and abusive behaviors toward older adults [40,41]. This association appears to operate largely through caregiver burden and impaired coping, whereby distress linked to anxiety and depressive symptoms heightens the risk of psychological abuse and neglect [40]. Taken together, the literature supports the GHQ‑12 as a useful indicator for identifying caregivers at elevated risk of elder abuse perpetration and highlights caregiver mental health as a key target for preventive interventions. The ZBI and GHQ-12 can therefore be used along with the STIEAC to determine the risk of elder abuse perpetration among caregivers.

The exploratory factor analysis revealed two primary components accounting for 71.45% of the variance. This largely unidimensional structure implies that a single, dominant latent construct, i.e., overall elder abuse perpetration accounts for the shared variance across the items encompassing dimensions of neglect, physical, psychological, financial, and sexual abuse. Rather than indicating that elder abuse is conceptually uniform however, this unidimensional structure shows that the assessed domains co-occur in a sufficient manner in practice for the overall measure to serve as a coherent indicator of the general abuse construct. This is consistent with the recognition in psychometric theory that items reflecting different manifestations of a complex phenomenon can load on a single factor when they represent intertwined processes [42,43]. Supportive evidence can be found in structured professional judgment instruments like the Short-Term Assessment of Risk and Treatability (START) which demonstrate that broad, multi-domain risk indicators can exhibit unidimensionality [43]. This concept has been described as essential unidimensionality where one general factor dominates despite the presence of meaningful secondary content which are neither irrelevant nor meant to be discarded [42]. A total score can therefore be used to capture general risk with the understanding that specific domains may contribute differentially. In the context of elder abuse, the emergence of a unidimensional factor supports the STIEAC’s utility for determining whether abuse perpetration is present overall, facilitating clear interpretation, robust reliability, and valid score summation, which are essential for screening, surveillance, and analytic purposes. At the same time, with regards to domain‑level identification unidimensionality implies that while individual items or subdomains can still be examined descriptively or clinically, their ability to function as independent psychometric subscales is limited, particularly for low‑base‑rate and highly sensitive domains such as sexual abuse. Nevertheless, the unidimensional structure of the STIEAC strengthens confidence in its ability to detect elder abuse perpetration in general considering the high Cronbach’s α. Confounding from separate domain-specific factors is minimized while enhancing the scale’s internal consistency. The unidimensionality however underscores the need for caution in interpretation of domain-specific scores as well as necessitating complementary qualitative, clinical or domain specific analysis when there is a need for identification of specific abuse types [44]. It is notable however that this structure validates the tool’s utility for detecting overall elder abuse perpetration in general screening contexts, as it allows for a reliable composite score that captures the essence of abuse without requiring domain-specific subscale interpretation, thereby supporting its practical application in epidemiological assessments of caregiver or perpetrator behavior.

The shift in the screening focus from the older person as the sole respondent to the caregiver as the potential perpetrator recognizes that some caregivers may unwittingly perpetrate abuse as a result of caregiver burden or lack of awareness. Caregiver self-reports allow caregivers to reflect on the circumstances, providing insight into the perpetration process, which may be helpful in the development of intervention strategies. The tool’s applicability extends beyond clinical settings, enabling community health practitioners and social workers to screen for elder abuse proactively. Using the STIEAC in instances where there is suspicion of elder abuse may enhance identification of abuse in community settings where elder abuse remains largely hidden owing to social stigma and underreporting. Its widespread use could inform targeted interventions, facilitate caregiver support programs, and influence policy formulation aimed at the protection of older persons. While caregiver‑focused screening may facilitate earlier identification of abusive dynamics and unmet support needs, it also introduces risks related to confidentiality, misclassification, and ethical issues related to reporting incidents and the logistics of who intervenes [44,45]. It is therefore imperative that whether the tool is used in clinical settings for research purposes or by community health workers, there should be transparent communication regarding the expectations and implications of potential findings. A collaborative and multidisciplinary approach can be established to ensure supportive, and informed responses within a public health framework. This will be particularly important in settings where mandatory reporting of abuse is expected and lines of communication with the responsible parties will need to be kept open prior to screening.

The reliance of the STIEAC on perpetrators’ self-reported abuse and neglect is acknowledged as a potential limitation, as this makes the tool subject to social desirability bias and potential underreporting, particularly given possible legal implications. However, self-report remains a common and pragmatic approach in perpetration research, especially when alternative data sources are unavailable or ethically challenging to obtain. These risks can be mitigated by the tool’s anonymity and non-judgmental wording to encourage disclosure among potential respondents. Further, the findings derived from this tool should be interpreted cautiously with consideration of the context.

Sexual abuse of older adults is often under‑detected, a limitation that is reflected in the design of existing elder abuse screening tools. Across validated instruments, sexual abuse is rarely operationalized as a discrete and systematically measured domain and is more commonly embedded within broader constructs such as coercion, vulnerability, or violations of personal rights. While a limited number of tools – notably the Elder Abuse Suspicion Index (EASI), Elder Assessment Instrument (EAI), Hwalek‑Sengstock Elder Abuse Screening Test (H‑S/EAST), and Family Members Mistreatment of Older Adults Screening Questionnaire (FAMOASQ) – demonstrate conceptual recognition of sexual abuse, many commonly used instruments focus on vulnerability, psychological abuse, caregiver behavior, or financial exploitation and do not explicitly assess sexual abuse [2,3,24]. In this context, reliance on brief, disclosure‑dependent screening approaches further constrains detection, particularly in settings shaped by stigma, dependency, cognitive impairment, and age‑related assumptions. Thus, the low internal consistency observed for the sexual abuse subscale of the STIEAC (Cronbach’s alpha = 0.03) can be viewed as reflecting both measurement limitations and the inherent sensitivity and cultural complexity of this construct, rather than an absence of relevance. It is also critical to note that despite the low internal consistency for this item, the overall Cronbach’s alpha for the tool drops from 0.90 to 0.85 when the item is deleted from the tool. Thus the sexual abuse domain remains an important aspect of the overall measurement of elder abuse. These findings underscore the need for cautious interpretation and support future methodological refinement through culturally informed qualitative item development and additional validation studies, rather than the exclusion of sexual abuse from elder abuse measurement frameworks.

Although the regional focus of the study may constitute a limitation to its generalizability to other Nigerian or African settings with differing cultural norms, the inclusion of other ethnic groups in the study area mitigates this limitation to some degree. The cross-sectional design limits inference about temporal patterns of caregiver abuse behaviors, emphasizing the need for longitudinal assessments. Future research should explore the tool’s predictive validity longitudinally, measure its effectiveness in various sociocultural contexts, and evaluate its integration into existing elder care frameworks. Future studies can also assess reducing bias and strengthening validity through triangulating self-report data with additional data sources. Training community health workers and social service providers to administer the STIEAC would optimize early detection efforts and potentially reduce the prevalence of elder abuse at the community level.

In conclusion, the STIEAC emerges as a promising, culturally sensitive instrument for identifying elder abuse perpetrated by caregivers in community contexts. Its comprehensive validation supports its potential as a vital screening resource, facilitating timely interventions and contributing to safeguarding the dignity and wellbeing of older persons in Nigeria and similar settings.

## Supporting information

S1_STIEAC~Tool The Screening Tool for Identifying Elder Abuse by Caregivers (STIEAC). (DOCX)

## References

[1] Cohen M. Screening tools for the identification of elder abuse. JCOM. 2011;18:261–70.

[2] Gallione C, Dal Molin A, Cristina FVB, Ferns H, Mattioli M, Suardi B. Screening tools for identification of elder abuse: a systematic review. J Clin Nurs. 2017;26:2154–76. 28042891 10.1111/jocn.13721

[3] Van Royen K, Van Royen P, De Donder L, Gobbens RJ. Elder Abuse Assessment Tools and Interventions for use in the Home Environment: a Scoping Review. Clin Interv Aging. 2020;15:1793–807. doi: 10.2147/CIA.S261877 33061330 PMC7533912

[4] Steinsheim G, Saga S, Olsen B, Broen HK, Malmedal W. Abusive episodes among home-dwelling persons with dementia and their informal caregivers: a cross-sectional Norwegian study. BMC Geriatr. 2022;22(1):852. doi: 10.1186/s12877-022-03569-4 36371161 PMC9655791

[5] Anetzberger GJ. Caregiving and elder abuse: A complex relationship. Handbook of interpersonal violence and abuse across the lifespan: A project of the National Partnership to End Interpersonal Violence Across the Lifespan (NPEIV). Springer International Publishing. 2021. p. 4633–57.

[6] Ho C, Wong SY, Chiu MM, Ho R, Ho CSH, Wong SY, et al. Global Prevalence of Elder Abuse: A Meta-analysis and Meta-regression. East Asian Arch Psychiatry. 2017. 28652497

[7] Carr D, Utz RL. Families in later life: a decade in review. J Marriage Fam. 2020;82(1):346–63. doi: 10.1111/jomf.12609 33633412 PMC7904069

[8] Pillemer K, Burnes D, Riffin C, Lachs MS. Elder Abuse: Global Situation, Risk Factors, and Prevention Strategies. Gerontologist. 2016;:S194-205. doi: 10.1093/geront/gnw004 PMC5291158 26994260

[9] Ranabhat P, Nikitara M, Latzourakis E, Constantinou CS. Effectiveness of Nurses’ Training in Identifying, Reporting and Handling Elderly Abuse: A Systematic Literature Review. Geriatrics (Switzerland). MDPI; 2022. doi: 10.3390/geriatrics7050108 PMC9602163 36286211

[10] Touza Garma C. Influence of health personnel’s attitudes and knowledge in the detection and reporting of elder abuse: An exploratory systematic review. Psychosocial Intervention. 2017;26(2):73–91. doi: 10.1016/j.psi.2016.11.001

[11] Mercier É, Nadeau A, Brousseau A-A, Émond M, Lowthian J, Berthelot S, et al. Elder Abuse in the Out-of-Hospital and Emergency Department Settings: A Scoping Review. Ann Emerg Med. 2020;75(2):181–91. doi: 10.1016/j.annemergmed.2019.12.011 31959308

[12] Fang J-H, Chen I-H, Lai H-R, Lee P-I, Miao N-F, Peters K, et al. Factors associated with nurses’ willingness to handle abuse of older people. Nurse Educ Pract. 2022;65:103497. doi: 10.1016/j.nepr.2022.103497 36347138

[13] Mazzotti MC, Scarcella E, D’Antone E, Fersini F, Salsi G, Ingravallo F, et al. Italian healthcare professionals’ attitude and barriers to mandatory reporting of elder abuse: An exploratory study. J Forensic Leg Med. 2019;63:26–30. doi: 10.1016/j.jflm.2019.02.007 30849694

[14] Mohit C, Harish D. Medico-legal approach in elderly abuse and neglect. INTERNATIONAL JOURNAL OF MEDICAL TOXICOLOGY & LEGAL MEDICINE. 2020;23(3and4):296–303. doi: 10.5958/0974-4614.2020.00081.9

[15] Ziminski Pickering CE, Rempusheski VF. Examining barriers to self-reporting of elder physical abuse in community-dwelling older adults. Geriatr Nurs. 2014;35(2):120–5. doi: 10.1016/j.gerinurse.2013.11.002 24341952

[16] Fang B, Yan E. Abuse of older persons with cognitive and physical impairments: comparing percentages across informants and operational definitions. J Interpers Violence. 2021;36:1682–98. 29295000 10.1177/0886260517742150

[17] Fang B, Li D, Yan E, Zhou Y, Yu Z, Hu J. Associated factors of discrepancy between older adults and their family caregivers in reporting elder abuse. J Clin Nurs. 2023;32:688–700. 35289011 10.1111/jocn.16283

[18] Ritchie CS, Roth DL, Allman RM. Living with an aging parent: “It was a beautiful invitation”. JAMA. 2011;306(7):746–53. doi: 10.1001/jama.2011.1163 21846856 PMC3817109

[19] Gimeno I, Val S, Cardoso Moreno MJ. Relation among Caregivers’ Burden, Abuse and Behavioural Disorder in People with Dementia. Int J Environ Res Public Health. 2021;18(3):1263. doi: 10.3390/ijerph18031263 33572503 PMC7908463

[20] Jaracz K, Grabowska-Fudala B, Górna K, Kozubski W. Caregiving burden and its determinants in Polish caregivers of stroke survivors. Arch Med Sci. 2014;10(5):941–50. doi: 10.5114/aoms.2014.46214 25395945 PMC4223139

[21] Kurasawa S, Yoshimasu K, Washio M, Fukumoto J, Takemura S, Yokoi K, et al. Factors influencing caregivers’ burden among family caregivers and institutionalization of in-home elderly people cared for by family caregivers. Environ Health Prev Med. 2012;17(6):474–83. doi: 10.1007/s12199-012-0276-8 22454030 PMC3493627

[22] Neuberg M, Meštrović T, Ribić R, Šubarić M, Canjuga I, Kozina G. Contrasting Vantage Points between Caregivers and Residents on the Perception of Elder Abuse and Neglect During Long-Term Care. Psychiatr Danub. 2019;31(Suppl 3):345–53. 31488751

[23] National Center on Elder Abuse. Research to Practice: Elder Abuse Screening Tools for Healthcare Professionals. http://eldermistreatment.usc.edu/wp-content/uploads/2016/10/Elder-Abuse-Screening-Tools-for-Healthcare-Professionals.pdf. 2016.

[24] Phelan A, Treacy MP. A review of elder abuse screening tools for use in the Irish context. Dublin. 2011.

[25] Artino AR Jr, La Rochelle JS, Dezee KJ, Gehlbach H. Developing questionnaires for educational research: AMEE Guide No. 87. Med Teach. 2014;36(6):463–74. doi: 10.3109/0142159X.2014.889814 24661014 PMC4059192

[26] Lachs M, Irene F, Psaty IR, Berman J, Caccamise PL, Cook AM, et al. Under the Radar: New York State Elder Abuse Prevalence Study. 2011.

[27] Marmoleji II. Elder Abuse in the Family in Spain. Valencia, Spain: Fundacion de la Comunitat Valenciana Para el Estudio de la Violencia (Centro Reina Sofia). 2008.

[28] Wilders G. ATLAS.ti - the knowledge workbench. Berlin: GmbH. 2015.

[29] Fereday J, Muir-Cochrane E. Demonstrating rigor using thematic analysis: A hybrid approach of inductive and deductive coding and theme development. Int J Qual Methods. 2006;5:80–92. doi: 10.1177/160940690600500107

[30] Ojifinni O, Adebayo E, Uchendu O. Insights into caregivers’ perspectives on abuse of older persons: a qualitative exploratory study in southwest Nigeria. BMJ Open. 2023;13(10):e070937. doi: 10.1136/bmjopen-2022-070937 37852773 PMC10603423

[31] Bédard M, Molloy DW, Squire L, Dubois S, Lever JA, O’Donnell M. The Zarit Burden Interview: A New Short and Screening Version. Gerontologist. 2001;41:652–7. doi: 10.1093/geront/41.5.652 11574710

[32] Chattat R, Cortesi V, Izzicupo F, Del Re ML, Sgarbi C, Fabbo A, et al. The Italian version of the Zarit Burden interview: a validation study. Int Psychogeriatr. 2011;23(5):797–805. doi: 10.1017/S1041610210002218 21205379

[33] Wallace M, Shelkey M. Katz Index of Independence in Activities of Daily Living (ADL). Best Practices in Nursing Care to Older Adults. 2007;21–2. 17390935

[34] Sterling M. General Health Questionnaire. J Physiother. 2011;106:481. doi: 10.1016/S1836-9553(11)70060-1 22093128

[35] Ojifinni OO, Uchendu OC. Validation and Reliability of the 12-item Zarit Burden Interview among Informal Caregivers of Elderly Persons in Nigeria. Arch Basic Appl Med. 2018;6(1):45–9. 29938213 PMC6010072

[36] Storey JE. Risk factors for elder abuse and neglect: A review of the literature. Aggress Violent Behav. 2020;50.

[37] Pabón-Poches DK, Ortiz-Ramirez HA, Cervantes-Henríquez ML, Barchelot Aceros LJ, Delgado Meza JA, Vallejo-Borda JA. Risk factors for elder abuse in the Global South: a case study in northeastern Colombia. Aging & Mental Health. 2025;30(1):30–43. doi: 10.1080/13607863.2025.2517676 40538376

[38] Letizia MA, Mark MK, Mohammed AM, Moses K, Scholastic A, Samuel M. Factors associated with elder abuse and neglect in rural Uganda: A cross-sectional study of community older adults attending an outpatient clinic. PLoS One. 2023;18. 10.1371/journal.pone.0280826 PMC9916607 36763620

[39] Özcan NK, Boyacıoğlu NE, Sertçelik E. Reciprocal Abuse: Elder Neglect and Abuse by Primary Caregivers and Caregiver Burden and Abuse in Turkey. Arch Psychiatr Nurs. 2017;31(2):177–82. doi: 10.1016/j.apnu.2016.09.011 28359430

[40] Gao X, Chen Y, Guo L, Sun F. Stressor, Coping, Resources and Potentially Harmful Behaviors by Family Caregivers of Cognitively Impaired Older Adults in China: The Mediation Role of Caregiver Burden. J Fam Viol. 2024;40(6):1157–67. doi: 10.1007/s10896-024-00691-2

[41] Cooper C, Barber J, Griffin M, Rapaport P, Livingston G. Effectiveness of START psychological intervention in reducing abuse by dementia family carers: randomized controlled trial. Int Psychogeriatr. 2016;28(6):881–7. doi: 10.1017/S1041610215002033 26652193

[42] Ziegler M, Hagemann D. Testing the unidimensionality of items. Psychological Assessment. 2015;31:231–7. doi: 10.1027/1015-5759/A000309

[43] Whittington R, Pollak C, Keski-Valkama A, Brown A, Haines-Delmont A, Bak J, et al. Unidimensionality of the Strengths and Vulnerabilities Scales in the Short-Term Assessment of Risk and Treatability (START). International Journal of Forensic Mental Health. 2022;21(2):175–84. doi: 10.1080/14999013.2021.1953193

[44] Saghafi A, Bahramnezhad F, Poormollamirza A, Dadgari A, Navab E. Examining the ethical challenges in managing elder abuse: a systematic review. J Med Ethics Hist Med. 2019;12:7. doi: 10.18502/jmehm.v12i7.1115 31346400 PMC6642445

[45] Beach SR, Carpenter CR, Rosen T, Sharps P, Gelles R. Screening and detection of elder abuse: Research opportunities and lessons learned from emergency geriatric care, intimate partner violence, and child abuse. J Elder Abuse Negl. 2016;28(4–5):185–216. doi: 10.1080/08946566.2016.1229241 27593945 PMC7339956

