# Supplementary material for: Development and validation of the screening tool for identifying elder abuse by caregivers (STIEAC)
Source: PLoS One. 2026 Jun 17;21(6):e0351005. doi: 10.1371/journal.pone.0351005 (PMC13274881; doi:10.1371/journal.pone.0351005)
Supplement: S1_STIEAC~Tool — (DOCX) [file pone.0351005.s001.docx]

# **The Screening Tool for Identifying Elder Abuse by Caregivers (STIEAC)**

| **Instructions**: Answer the following questions concerning your care of the older person for whom you are responsible using the codes:  ***1*** *= Once in the past year*  ***2*** *= Twice in the past year*  ***3*** *= Approximately 5 times in the past year*  ***4*** *= 6-10 times in the past year*  ***5*** *= More than 10 times in the past year*  ***6*** *= Not in the past year, but it has happened before*  ***7*** *= This has never happened* | | | | |
| --- | --- | --- | --- | --- |
| **Item** | **Abuse type** | **Statement** | **Code** |  |
|  | Neglect | Would you say you have ever been indifferent to his/her nutritional desires? |  |  |
|  |  | Do you think you have ever been unconcerned about his/her clothing? |  |  |
|  |  | Would you say you have ever been unconcerned regarding his/her hygiene? |  |  |
|  | Physical | Have you ever restricted him/her with some mechanism that prevented them from moving about freely, locked them up or put them in a chair from which they could not get up without help? |  |  |
|  |  | Have you ever given him/her non-prescribed pharmaceutical drugs for any reason at all? |  |  |
|  | Psychological/Emotional | Have you ever failed to care for their affective or emotional needs? |  |  |
|  |  | Have you ever intimidated or threatened them in any way? |  |  |
|  | Financial | Have you ever forced them to sign documents or change their will? |  |  |
|  |  | Have you ever faked their signature? |  |  |
|  | Sexual | Have you ever engaged in sexual activity with them without their consent? |  |  |
