# Supplementary material for: Development and validation of the screening tool for identifying elder abuse by caregivers (STIEAC)
Source: PLoS One. 2026 Jun 17;21(6):e0351005. doi: 10.1371/journal.pone.0351005 (PMC13274881; doi:10.1371/journal.pone.0351005)
Supplement: Attachment — Submitted filename: Review 27.2.2026.docx [file pone.0351005.s002.docx]

**Review Summary**

Manuscript ID: PONE-D-25-61417
Journal: PLOS ONE

This study described in the manuscript elaborating the development and validation of the Screening Tool for Identifying Elder Abuse by Caregivers (STIEAC) in Southwest Nigeria gives a new perspective of looking at elder abuse research by shifting the focus from victim-reported abuse to caregiver self-reported perpetration. The study being conducted in the community settings is a huge strength as it takes into consideration the contextual importance and adaptability component. This work is the need of the hour especially in the Sub-Saharan Africa wherein the use of adapted tools is not prevalent and hence the results are not reliable.

The use of exploratory sequential mixed methods design is another strength of the study as it used a structured questionnaire development framework (based on work by Anthony R. Artino Jr.) to start with and further integrated qualitative data to ensure contextual relevance, expert review for content validity, and psychometric testing including internal consistency, exploratory factor analysis, and construct validity.

With the Cronbach’s α = 0.90, the overall internal consistency of the tool is strong while the correlations with caregiver burden and mental health measures provide preliminary support for construct validity.

However, I suggest major revision due to several important concerns outlined below:

1. **The sample size** (n=106) is a major limiting factor for a strong psychometric validation
2. The **sexual abuse domain** on the questionnaire has only 1 item and that too with very poor reliability
3. There is a serious data reporting error in the **standard deviation of the sexual abuse** item.
4. The **factor analysis** suggests a largely unidimensional structure inspite of five abuse domains being conceptually proposed
5. Substantial explanation is necessary for the **correlations with GHQ-12**
6. An important aspect of the **social desirability bias** which is always inherent in the self-reported perpetration measures has not been satisfactorily taken into consideration.

In summary, the manuscript will add to an important and pressing concern area of elder abuse screening research especially in the most needed community settings. But, extensive explanation is required about:

a) errors in reporting
b) psychometric findings
c) methodological limitations

**Recommendation: Major Revision.**
