# Supplementary material for: Development and validation of the screening tool for identifying elder abuse by caregivers (STIEAC)
Source: PLoS One. 2026 Jun 17;21(6):e0351005. doi: 10.1371/journal.pone.0351005 (PMC13274881; doi:10.1371/journal.pone.0351005)
Supplement: Attachment — Submitted filename: Response to reviewers.docx [file pone.0351005.s004.docx]

**Response to Review Comments**

We are grateful to the reviewers for the comments provided to improve the quality of our manuscript. Below is our response to the review comments.

| **S/N** | **Review comment** | **Response** | **Section/Page** |
| --- | --- | --- | --- |
|  | **Reviewers' comments** |  |  |
|  | **Reviewer #1** |  |  |
| 1. | This is a very interesting study with clinical applications. However, few things should be addressed - at least mentioned the limitation sections. | Thank you. |  |
| 2. | The tool is based on self-report abuse and neglect by perpetrators. There is a high risk of bias, participants to not disclose for legal issues. | This potential is recognised, however, self-report may be the only option in instances of suspicion and the tool’s non-judgemental wording may mitigate this potential bias. This has been acknowledged in the discussion. | Lines 284 – 291 |
| 3. | Several ethical considerations should be taken account regarding how the tool should be used, by whom, and what will happen next to the person who might revealed actions of abuse. | This has been highlighted and possible responses described in the discussion. | Lines 340 - 349 |
| 4. | The score of Guttman split-half correlation (0.713) is the minimum score for acceptable reliability. This should be reported. | This has been inserted into the results. | Line 215 |
| 5. | The low reliability and consistency for sexual abuse could have been explained more in the discussion, comparing evidence based from relevant studies. | This has been highlighted in the results. | Line 301 – 318 |
| 6. | Although in study design 7 steps were conducting. There could be results for each of this step, demonstrating the process better. | The outcome of the steps is outlined in the tool validation section within the methodology. | Line 155 – 183 |
| 7. | There are no results from the literature review listing the existing tools. | This has been provided in the introduction. | Lines 80 – 95 |
|  | **Reviewer #2** |  |  |
| 8. | The research is interesting as the researchers tried to develop and validate the Screening Tool for identifying the elder abuse by the caregivers. But the major concern is only 106 quantitative interviews are conducted within a particular ethnic in a particular region of Nigeria. This raises the question on the validity of the statistical analysis conducted. Further, regional focus of the research also questions the application of such tool in other cultural settings. Although this was indicated in the limitation of the study, it is suggested to conduct the study in other cultural settings (may be within Africa with other ethnic groups) to find the applicability of this tool. Otherwise, it will be difficult to have any policy implication out of this research. | The 106 interviews made up the pilot test of the questionnaire which initially included 20 questions. Analysis of the pilot test data included principal component analysis with removal of items whose factor loadings were <0.4 leaving the 10 items that were included in a larger survey including 1119 participants. The reliability analysis was repeated in this larger survey and has now been presented in the manuscript. | Line 169 – 180, 221 – 224, 239 – 249 |
|  | **Reviewer #3** |  |  |
| 9. | The sample size (n=106) is a major limiting factor for a strong psychometric validation | This comment has been addressed in point 14. |  |
| 10. | The sexual abuse domain on the questionnaire has only 1 item and that too with very poor reliability. | The poor reliability of the sexual abuse domain is recognised and from literature, it is shown that this domain is one that is difficult to measure due to cultural issues. Previous tools are either silent on this or only recognise it as a concept integrated into other aspects of abuse. This has been highlighted in detail in the discussion. | Line 305 – 326 |
| 11. | There is a serious data reporting error in the standard deviation of the sexual abuse item. | Thank you. It was a typographical error and has been corrected. | Table 5 |
| 12. | The factor analysis suggests a largely unidimensional structure in spite of five abuse domains being conceptually proposed | An explanation has been provided for this in tandem with existing literature in the discussion. | 33Lines 301 – 334 |
| 13. | Substantial explanation is necessary for the correlations with GHQ-12 | This has been added. | Lines 298 – 308 |
| 14. | An important aspect of the social desirability bias which is always inherent in the self-reported perpetration measures has not been satisfactorily taken into consideration. | This has been addressed in the discussion. | Lines 355 - 362 |
