# Supplementary material for: Development and validation of the screening tool for identifying elder abuse by caregivers (STIEAC)
Source: PLoS One. 2026 Jun 17;21(6):e0351005. doi: 10.1371/journal.pone.0351005 (PMC13274881; doi:10.1371/journal.pone.0351005)
Supplement: Peer review history

# Peer review: Decision Letter 0

**DOI:** 10.1371/journal.pone.0351005.r001

**Authors:** Shivanand Kattimani

5 Mar 2026

PONE-D-25-61417

Development and validation of the screening tool for identifying elder abuse by caregivers (STIEAC)

PLOS One

Dear Dr. Ojifinni,

Thank you for submitting your manuscript to PLOS ONE. After careful consideration, we feel that it has merit but does not fully meet PLOS ONE’s publication criteria as it currently stands. Therefore, we invite you to submit a revised version of the manuscript that addresses the points raised during the review process.

Please submit your revised manuscript by Apr 19 2026 11:59PM. If you will need more time than this to complete your revisions, please reply to this message or contact the journal office at plosone@plos.org. When you're ready to submit your revision, log on to https://www.editorialmanager.com/pone/ and select the 'Submissions Needing Revision' folder to locate your manuscript file.

Please include the following items when submitting your revised manuscript:

- A letter that responds to each point raised by the academic editor and reviewer(s). You should upload this letter as a separate file labeled 'Response to Reviewers'.
- A marked-up copy of your manuscript that highlights changes made to the original version. You should upload this as a separate file labeled 'Revised Manuscript with Track Changes'.
- An unmarked version of your revised paper without tracked changes. You should upload this as a separate file labeled 'Manuscript'.

If you would like to make changes to your financial disclosure, please include your updated statement in your cover letter. Guidelines for resubmitting your figure files are available below the reviewer comments at the end of this letter.

If applicable, we recommend that you deposit your laboratory protocols in protocols.io to enhance the reproducibility of your results. Protocols.io assigns your protocol its own identifier (DOI) so that it can be cited independently in the future. For instructions see: https://journals.plos.org/plosone/s/submission-guidelines#loc-laboratory-protocols. Additionally, PLOS ONE offers an option for publishing peer-reviewed Lab Protocol articles, which describe protocols hosted on protocols.io. Read more information on sharing protocols at https://plos.org/protocols?utm_medium=editorial-email&utm_source=authorletters&utm_campaign=protocols.

We look forward to receiving your revised manuscript.

Kind regards,

Shivanand Kattimani

Academic Editor

PLOS One

Journal Requirements:

When submitting your revision, we need you to address these additional requirements.

1. Please ensure that your manuscript meets PLOS ONE's style requirements, including those for file naming. The PLOS ONE style templates can be found at

https://journals.plos.org/plosone/s/file?id=wjVg/PLOSOne_formatting_sample_main_body.pdf and https://journals.plos.org/plosone/s/file?id=ba62/PLOSOne_formatting_sample_title_authors_affiliations.pdf

2.   Your ethics statement should only appear in the Methods section of your manuscript. If your ethics statement is written in any section besides the Methods, please delete it from any other section.

3. We note that you have indicated that there are restrictions to data sharing for this study. For studies involving human research participant data or other sensitive data, we encourage authors to share de-identified or anonymized data. However, when data cannot be publicly shared for ethical reasons, we allow authors to make their data sets available upon request. For information on unacceptable data access restrictions, please see http://journals.plos.org/plosone/s/data-availability#loc-unacceptable-data-access-restrictions.

Before we proceed with your manuscript, please address the following prompts:

a) If there are ethical or legal restrictions on sharing a de-identified data set, please explain them in detail (e.g., data contain potentially identifying or sensitive patient information, data are owned by a third-party organization, etc.) and who has imposed them (e.g., a Research Ethics Committee or Institutional Review Board, etc.). Please also provide contact information for a data access committee, ethics committee, or other institutional body to which data requests may be sent.

b) If there are no restrictions, please upload the minimal anonymized data set necessary to replicate your study findings to a stable, public repository and provide us with the relevant URLs, DOIs, or accession numbers. Please see http://www.bmj.com/content/340/bmj.c181.long for guidelines on how to de-identify and prepare clinical data for publication. For a list of recommended repositories, please see https://journals.plos.org/plosone/s/recommended-repositories. You also have the option of uploading the data as Supporting Information files, but we would recommend depositing data directly to a data repository if possible.

Please update your Data Availability statement in the submission form accordingly.

4. Please amend your authorship list in your manuscript file to include author Oludoyinmola Ojifinni.

5. Please amend the manuscript submission data (via Edit Submission) to include author Oludoyinmola Omobolade OJIFINNI.

6. Please include captions for your Supporting Information files at the end of your manuscript, and update any in-text citations to match accordingly. Please see our Supporting Information guidelines for more information: http://journals.plos.org/plosone/s/supporting-information.

Reviewers' comments:

Reviewer's Responses to Questions

**Comments to the Author**

1. Is the manuscript technically sound, and do the data support the conclusions?

The manuscript must describe a technically sound piece of scientific research with data that supports the conclusions. Experiments must have been conducted rigorously, with appropriate controls, replication, and sample sizes. The conclusions must be drawn appropriately based on the data presented.

Reviewer #1: Yes

Reviewer #2: Partly

Reviewer #3: Partly

**********

2. Has the statistical analysis been performed appropriately and rigorously?

Reviewer #1: Yes

Reviewer #2: Yes

Reviewer #3: No

**********

3. Have the authors made all data underlying the findings in their manuscript fully available?

The PLOS Data policy requires authors to make all data underlying the findings described in their manuscript fully available without restriction, with rare exception (please refer to the Data Availability Statement in the manuscript PDF file). The data should be provided as part of the manuscript or its supporting information, or deposited to a public repository. For example, in addition to summary statistics, the data points behind means, medians and variance measures should be available. If there are restrictions on publicly sharing data—e.g. participant privacy or use of data from a third party—those must be specified.

Reviewer #1: Yes

Reviewer #2: Yes

Reviewer #3: No

**********

4. Is the manuscript presented in an intelligible fashion and written in standard English?

PLOS ONE does not copyedit accepted manuscripts, so the language in submitted articles must be clear, correct, and unambiguous. Any typographical or grammatical errors should be corrected at revision, so please note any specific errors here.

Reviewer #1: Yes

Reviewer #2: Yes

Reviewer #3: Yes

**********

5. Review Comments to the Author

Please use the space provided to explain your answers to the questions above. You may also include additional comments for the author, including concerns about dual publication, research ethics, or publication ethics. (Please upload your review as an attachment if it exceeds 20,000 characters)

Reviewer #1: This is a very interesting study with clinical applications.

However few things should be addressed - at least mentioned the limitation sections:

The tool is based on self-report abuse and neglect by perpetrators. There is a high risk of bias, participants to not disclose for legal issues. Several ethical considerations should be taken account regarding how the tool should be used, by whom, and what will happen next to the person who might revealed actions of abuse.

The score of Guttman split-half correlation (0.713) is the minimum score for acceptable reliability. This should be reported.

The low reliability and consistency for sexual abuse could have been explained more in the discussion, comparing evidence based from relevant studies.

Although in study design 7 steps were conducting. There could be results for each of this step, demonstrating the process better.

There are no results from the literature review listing the existing tools.

Reviewer #2: The research is interesting as the researchers tried to develop and validate the Screening Tool for identifying the elder abuse by the caregivers. But the major concern is only 106 quantitative interviews are conducted within a particular ethnic in a particular region of Nigeria. This raises the question on the validity of the statistical analysis conducted. Further, regional focus of the research also questions the application of such tool in other cultural settings. Although this was indicated in the limitation of the study, it is suggested to conduct the study in other cultural settings (may be within Africa with other ethnic groups) to find the applicability of this tool. Otherwise, it will be difficult to have any policy implication out of this research.

Reviewer #3: I suggest major revision due to several important concerns outlined below:

1. The sample size (n=106) is a major limiting factor for a strong psychometric validation

2. The sexual abuse domain on the questionnaire has only 1 item and that too with very poor reliability

3. There is a serious data reporting error in the standard deviation of the sexual abuse item.

4. The factor analysis suggests a largely unidimensional structure inspite of five abuse domains being conceptually proposed

5. Substantial explanation is necessary for the correlations with GHQ-12

6. An important aspect of the social desirability bias which is always inherent in the self-reported perpetration measures has not been satisfactorily taken into consideration.

In summary, the manuscript will add to an important and pressing concern area of elder abuse screening research especially in the most needed community settings. But, extensive explanation is required about:

a) errors in reporting

b) psychometric findings

c) methodological limitations

**********

6. PLOS authors have the option to publish the peer review history of their article (what does this mean?). If published, this will include your full peer review and any attached files.

If you choose “no”, your identity will remain anonymous but your review may still be made public.

**Do you want your identity to be public for this peer review?**  For information about this choice, including consent withdrawal, please see our Privacy Policy.

Reviewer #1: No

Reviewer #2: **Yes:**  Dr. JAYANTA KUMAR BASU

Reviewer #3: **Yes:**  Amruta Bandal

**********

[NOTE: If reviewer comments were submitted as an attachment file, they will be attached to this email and accessible via the submission site. Please log into your account, locate the manuscript record, and check for the action link "View Attachments". If this link does not appear, there are no attachment files.]

To ensure your figures meet our technical requirements, please review our figure guidelines: https://journals.plos.org/plosone/s/figures

You may also use PLOS’s free figure tool, NAAS, to help you prepare publication quality figures: https://journals.plos.org/plosone/s/figures#loc-tools-for-figure-preparation.

NAAS will assess whether your figures meet our technical requirements by comparing each figure against our figure specifications.

Attachment Submitted filename: Review 27.2.2026.docx

# Author comment: Author response to Decision Letter 1

**DOI:** 10.1371/journal.pone.0351005.r002

6 May 2026

1. Please ensure that your manuscript meets PLOS ONE's style requirements, including those for file naming.

Done.

2. Your ethics statement should only appear in the Methods section of your manuscript. If your ethics statement is written in any section besides the Methods, please delete it from any other section.

The ethics statement is only in the methods section. Pages 9 – 10, lines 183 – 191

3. We note that you have indicated that there are restrictions to data sharing for this study. For studies involving human research participant data or other sensitive data, we encourage authors to share de-identified or anonymized data. However, when data cannot be publicly shared for ethical reasons, we allow authors to make their data sets available upon request. Before we proceed with your manuscript, please address the following prompts:

a) If there are ethical or legal restrictions on sharing a de-identified data set, please explain them in detail (e.g., data contain potentially identifying or sensitive patient information, data are owned by a third-party organization, etc.) and who has imposed them (e.g., a Research Ethics Committee or Institutional Review Board, etc.). Please also provide contact information for a data access committee, ethics committee, or other institutional body to which data requests may be sent.

b) If there are no restrictions, please upload the minimal anonymized data set necessary to replicate your study findings to a stable, public repository and provide us with the relevant URLs, DOIs, or accession numbers.

Please update your Data Availability statement in the submission form accordingly.

We appreciate the journal’s emphasis on data sharing and transparency. However, although de-identified, the dataset includes location-based indicators (the rural and urban wards and LGA names) as they informed part of the analysis for comparison of the differences between urban and rural dwelling populations. When combined with other variables, the risk of re-identification of participants is increased raising concerns about stigma and confidentiality. This potentially puts the study participants at risk of identification; hence, we cannot ethically justify public availability of the dataset. Further, the consent process included assuring participants of the confidentiality of their data and that the data would not be shared publicly beyond the research team. This was part of the protocol for which the original ethics clearance was obtained from the Oyo State Ministry of Health Ethical Committee (REF: AD13/479/974). Due to these ethical constraints, we cannot upload the data to a public repository. The data will however be made available to interested researchers upon reasonable request subject to review and approval by the Oyo State Ministry of Health Ethical Committee and adherence to a data-use agreement that ensures confidentiality and responsible use.

The data availability statement has been modified to state the following “The data availability statement has been modified to state the following “The data underlying the findings of this study contain sensitive information on elder abuse perpetration and participant location (rural vs. urban) which were collected under an informed consent process that emphasized confidentiality. The original ethics approval did not include provisions for public data sharing. Therefore, the dataset cannot be made publicly available. Data are available upon application to and approval by the Oyo State Ministry of Health Ethical Committee and adherence to a data‑use agreement that ensures confidentiality and responsible use. The ethics committee can be contacted vie email: oyostateethicsreviewcommittee@gmail.com. The project details are available at https://osf.io/zw83a/overview?view_only=db27644f2fd340e8a1594430075d0562 the dataset is also uploaded privately on figshare via this link https://doi.org/10.6084/m9.figshare.32112454.”

Please amend your authorship list in your manuscript file to include author Oludoyinmola Ojifinni. Please amend the manuscript submission data (via Edit Submission) to include author Oludoyinmola Omobolade OJIFINNI.

This has been amended. Both names refer to the same person.

Please include captions for your Supporting Information files at the end of your manuscript, and update any in-text citations to match accordingly. Please see our Supporting Information guidelines for more information: http://journals.plos.org/plosone/s/supporting-information.

This has been done.

Attachment Submitted filename: Response to reviewers.docx

# Peer review: Decision Letter 1

**DOI:** 10.1371/journal.pone.0351005.r003

**Authors:** Shivanand Kattimani

22 May 2026

Development and validation of the screening tool for identifying elder abuse by caregivers (STIEAC)

PONE-D-25-61417R1

Dear Dr. Ojifinni,

We’re pleased to inform you that your manuscript has been judged scientifically suitable for publication and will be formally accepted for publication once it meets all outstanding technical requirements.

Within one week, you’ll receive an e-mail detailing the required amendments. When these have been addressed, you’ll receive a formal acceptance letter and your manuscript will be scheduled for publication.

An invoice will be generated when your article is formally accepted. Please note, if your institution has a publishing partnership with PLOS and your article meets the relevant criteria, all or part of your publication costs will be covered. Please make sure your user information is up-to-date by logging into Editorial Manager at Editorial Manager® and clicking the ‘Update My Information' link at the top of the page. For questions related to billing, please contact billing support.

If your institution or institutions have a press office, please notify them about your upcoming paper to help maximize its impact. If they’ll be preparing press materials, please inform our press team as soon as possible -- no later than 48 hours after receiving the formal acceptance. Your manuscript will remain under strict press embargo until 2 pm Eastern Time on the date of publication. For more information, please contact onepress@plos.org.

Kind regards,

Shivanand Kattimani

Academic Editor

PLOS One

Reviewers' comments:

Reviewer's Responses to Questions

**Comments to the Author**

1. If the authors have adequately addressed your comments raised in a previous round of review and you feel that this manuscript is now acceptable for publication, you may indicate that here to bypass the “Comments to the Author” section, enter your conflict of interest statement in the “Confidential to Editor” section, and submit your "Accept" recommendation.

Reviewer #1: (No Response)

Reviewer #2: All comments have been addressed

**********

2. Is the manuscript technically sound, and do the data support the conclusions?

The manuscript must describe a technically sound piece of scientific research with data that supports the conclusions. Experiments must have been conducted rigorously, with appropriate controls, replication, and sample sizes. The conclusions must be drawn appropriately based on the data presented.

Reviewer #1: Yes

Reviewer #2: Yes

**********

3. Has the statistical analysis been performed appropriately and rigorously?

Reviewer #1: Yes

Reviewer #2: Yes

**********

4. Have the authors made all data underlying the findings in their manuscript fully available?

The PLOS Data policy requires authors to make all data underlying the findings described in their manuscript fully available without restriction, with rare exception (please refer to the Data Availability Statement in the manuscript PDF file). The data should be provided as part of the manuscript or its supporting information, or deposited to a public repository. For example, in addition to summary statistics, the data points behind means, medians and variance measures should be available. If there are restrictions on publicly sharing data—e.g. participant privacy or use of data from a third party—those must be specified.

Reviewer #1: No

Reviewer #2: Yes

**********

5. Is the manuscript presented in an intelligible fashion and written in standard English?

PLOS ONE does not copyedit accepted manuscripts, so the language in submitted articles must be clear, correct, and unambiguous. Any typographical or grammatical errors should be corrected at revision, so please note any specific errors here.

Reviewer #1: Yes

Reviewer #2: Yes

**********

6. Review Comments to the Author

Please use the space provided to explain your answers to the questions above. You may also include additional comments for the author, including concerns about dual publication, research ethics, or publication ethics. (Please upload your review as an attachment if it exceeds 20,000 characters)

Reviewer #1: (No Response)

Reviewer #2: Comments suggested were mostly addressed or incorporated. However, the authors explained the limitations of this research to other cultural norms and in the other regions or countries.

**********

7. PLOS authors have the option to publish the peer review history of their article (what does this mean?). If published, this will include your full peer review and any attached files.

If you choose “no”, your identity will remain anonymous but your review may still be made public.

**Do you want your identity to be public for this peer review?**  For information about this choice, including consent withdrawal, please see our Privacy Policy.

Reviewer #1: No

Reviewer #2: **Yes:**  Dr. JAYANTA KUMAR BASU

**********

# Editor report: Acceptance letter

**DOI:** 10.1371/journal.pone.0351005.r004

**Authors:** Shivanand Kattimani

PONE-D-25-61417R1

PLOS One

Dear Dr. Ojifinni,

I'm pleased to inform you that your manuscript has been deemed suitable for publication in PLOS One. Congratulations! Your manuscript is now being handed over to our production team.

At this stage, our production department will prepare your paper for publication. This includes ensuring the following:

* All references, tables, and figures are properly cited

* All relevant supporting information is included in the manuscript submission,

* There are no issues that prevent the paper from being properly typeset

You will receive further instructions from the production team, including instructions on how to review your proof when it is ready. Please keep in mind that we are working through a large volume of accepted articles, so please give us a few days to review your paper and let you know the next and final steps.

Lastly, if your institution or institutions have a press office, please let them know about your upcoming paper now to help maximize its impact. If they'll be preparing press materials, please inform our press team within the next 48 hours. Your manuscript will remain under strict press embargo until 2 pm Eastern Time on the date of publication. For more information, please contact onepress@plos.org.

You will receive an invoice from PLOS for your publication fee after your manuscript has reached the completed accept phase. If you receive an email requesting payment before acceptance or for any other service, this may be a phishing scheme. Learn how to identify phishing emails and protect your accounts at https://explore.plos.org/phishing.

If we can help with anything else, please email us at customercare@plos.org.

Thank you for submitting your work to PLOS ONE and supporting open access.

Kind regards,

PLOS ONE Editorial Office Staff

on behalf of

Dr. Shivanand Kattimani

Academic Editor

PLOS One
